# Health Justice Through the Lens of Power

**DOI:** 10.1017/jme.2023.5

**Published:** 2022

**Authors:** Jamila Michener

**Affiliations:** 1. CORNELL UNIVERSITY, ITHICA, NY, USA.

**Keywords:** Health Justice, Power, Politics

## Abstract

Health justice is an aspirational north star for scholars, practitioners, and anyone who refuses to accept the status quo of profound inequity. But what does health justice mean? How ought we conceptualize it? There is no correct answer to these questions, but any robust rendering of health justice must account for power and politics. This article posits that the path to health justice requires political struggle taking (at least) two forms: (1) building power and (2) breaking power. Building power for health justice means cultivating the political capacity of people who are disproportionately harmed by health inequity, and who therefore have the most at stake. Breaking power involves weakening and destabilizing the economic and political forces that perpetuate health inequity. By surfacing and elaborating these crucial modes of political struggle, this article points to a way forward for achieving health justice.

“Power concedes nothing without a demand. It never has and it never will.”

*Frederick Douglass*

It is difficult to deny the stark and painful realities of health injustice in the United States. Consider just a few relevant patterns. On average, Americans who are racialized as Black and American Indian/Alaska Native (AIAN) live fewer years than hose who are racialized as White, they are more likely to die from treatable conditions, and they are at higher risk of mortality throughout the life course — from infancy to adulthood.^1^ These outcomes are not the result of genetic or biological differences.^2^ To the contrary, race is a “social fact” with material repercussions inscribed via economic, political, and other structural processes.^3^ COVID-19 exposed such processes with devastating clarity. In the wake of the pandemic, average life expectancies for people racialized as Black, Latina/o, and AIAN fell sharply.^4^ Though striking, these outcomes barely touch the surface of a much deeper well of health disparities that span a full gamut of (often intersecting) categories of social difference (e.g., race, gender, and class). Such disparities corrode the fabric of U.S. society and compromise everyone’s well-being. Importantly, the unequal health status of marginalized populations is primarily a product of systemic forces, not individual behavior. Indeed, there is growing recognition that health inequity is rooted in structural factors such as racism and poverty.^5^ Identifying and understanding such structural determinants is crucial. But ultimately, the highest imperative is to change them. Empirically chronicling health disparities is of limited value unless we also chart a course towards health justice — a world where the structural positioning of people and communities (vis-à-vis ascriptive categories of difference) does not dictate their health and well-being.

## Health Justice and Power

Health justice is both an outcome and a process. As an outcome, health justice reflects a vision — defined by and with people and communities in specific contexts — for what every person and community should have, irrespective of race, ethnicity, class, gender, or any other dimension of socially inscribed difference.^6^ As a process, health justice entails transforming existing economic and political institutions to make them more inclusive, responsive, and accountable, particularly in relation to the needs and demands of those who are consistently and systematically marginalized.^7^ In this way, health justice is closely aligned with health equity. Consider the widely cited definition of health equity offered by Braveman et al. (2018):

Health equity means that everyone has a fair and just opportunity to be as healthy as possible. Achieving this requires removing obstacles to health such as poverty and discrimination and their consequences, which include powerlessness and lack of access to good jobs with fair pay; quality education, housing, and health care; and safe environments.^8^

This conceptualization of health equity attends to both outcome and process, end and means. Ensuring “a fair and just opportunity to be as healthy as possible” is the end, while eradicating poverty and securing fair pay, quality education, housing, health care, and safe environments are the means of getting there.

Identifying and understanding such structural determinants is crucial. But ultimately, the highest imperative is to change them. Empirically chronicling health disparities is of limited value unless we also chart a course towards health justice — a world where the structural positioning of people and communities (vis-à-vis categories of difference) does not dictate their health and well-being.

Given the historical and contemporary political economy of the United States, these ends and means both seem improbable.^9^ Current political and economic conditions (e.g., massive income and wealth inequality, disproportionate political power in the hands of economic elites) are advancing neither health equity nor health justice.^10^ Mapping the route between these prevailing realities and the aspiration of health justice demands close attention to the role of power. A focus on power pushes us to interrogate the political processes that make profound health inequities possible. In the pages that follow, I make a case for viewing health justice through the lens of political power. Along the way, I argue that achieving health justice requires *both* building power among those who are most deeply affected (corporeally and materially) by health inequity *and* breaking the power of those who are accruing (economic and political) gains from the status quo of health inequity.

## Conceptualizing Political Power

Though there is a significant corpus of research focused on health politics, a comparatively small subset of that work directly attends to power.^11^ Similarly, scholars studying health equity remain insufficiently concerned with power. This is understandable. Power is an amorphous concept that can appear impractically abstract when juxtaposed with the tangible facts of health inequity in the United States. The task of statistically measuring health disparities is markedly more tractable than the project of assessing the ebb, flow, and consequences of power. Moreover, power analyses implicate a range of actors and processes (e.g., health providers, hospital systems, academic institutions, economic practices, etc.) in ways that may be incriminating or simply difficult to grapple with, even for ostensible supporters of health equity. For these reasons, power often remains unnoticed and unspoken. This article is rooted in a core claim: drawing power out of the shadows and into the light can help illuminate a path towards health justice.

Power has been widely studied and theorized.^12^ There is no single or best way to define it. Instead, ideas about power are contingent on the context and purpose for which they are deployed. To ground our conceptualization of power in relation to health equity and justice, I follow Rosino in defining political power as “the capacity to influence social and structural conditions…through the state and political sphere.”^13^ This capacity-oriented view of power emphasizes the “power to” affect outcomes.^14^ Such power can be exercised on the individual or group levels and it “hinges on leveraging the capacities afforded via political systems.”^15^ Such leveraging happens among a wide range of actors who — often intentionally and sometimes unintentionally —“navigate political environments in ways that move the needle towards their desired outcomes.”^16^ For example, in the case of Medicaid expansion via the Affordable Care Act, influential actors ranged from conservative political networks to the hospital industry to business and professional lobbyists to public health advocates. These and other actors, sometimes in coalition, influenced whether states decided to expand Medicaid.^17^ Similarly, for all policies (including those structuring health) some set of actors use the political system to influence policy outputs. This means that policy changes essential for realizing health justice are only possible if people and groups seeking to eradicate obstacles to health (e.g., “poverty and discrimination…powerlessness and lack of access to good jobs with fair pay; quality education, housing, and health care; and safe environments”) have significant power.^18^ Even further, such people and groups must have greater influence over policy than those who oppose them.

**Figure 1:**
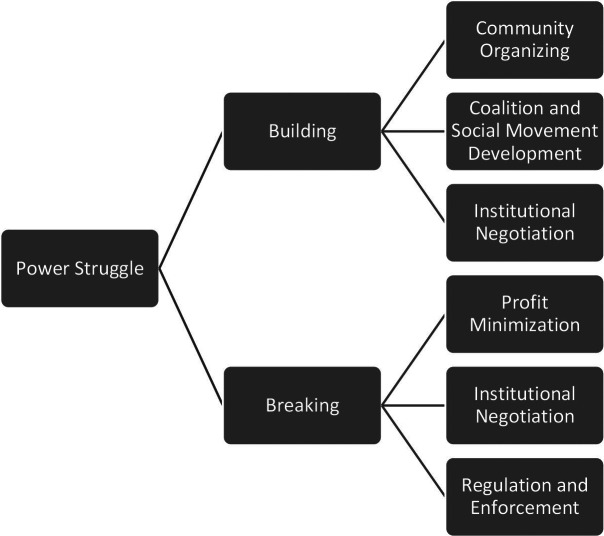
Building and Breaking Power

Political power in the United States is rarely configured this way. Instead, health outcomes and associated policies are often influenced by economic and political elites whose interests are (either explicitly or implicitly) antithetical to the aims of health justice.^19^ This suggests that health justice necessitates substantial redistribution of power. As Frederick Douglass aptly declared, “This struggle may be a moral one, or it may be a physical one…but it must be a struggle. Power concedes nothing without a demand. It never has and it never will.”^20^ Since historical and contemporary alignments of power have produced and perpetuated the status quo of health inequity, altering this trajectory will involve struggles for power. Such struggle can take (at least) two forms: 1) building power among those who are most affected by health inequity 2) breaking the power of interests that undermine health equity (see Figure 1).

## Building Power

Building power for health justice means cultivating the political capacity of people with the most at stake, those who are disproportionately harmed by health injustice. This constituency includes people racialized as Black, AIAN, and Latino, people living in poverty, disabled people, undocumented people, and anyone whose health is imperiled under present conditions. Figure 1 is a stylized representation of three reinforcing mechanisms for building power among such heterogenous groups: 1) community organizing; 2) coalition and social movement seeding and development; and 3) strategic institutional negotiation. In the rest of this section, I briefly elaborate on each.

Community organizing is an imperative element of building power.^21^ Organizations are like a “prism that refracts the actions of a constituency into political power.”^22^ The harms of health inequity are unduly borne by communities facing substantial barriers to collective action. Effective community organizing lowers those barriers by helping people to identify the systemic failures underlying their individual problems. In this way, community organizing creates opportunities for collective action in response to such failures.^23^ Organizing is a fundamental mechanism for channeling the “people power” that otherwise often lies dormant within marginalized communities.

The development and seeding of coalitions, alliances, and social movements is another essential dimension of power building.^24^ These formations facilitate the scaling and amplification of organizing efforts through networks of actors who share common goals, organizational links, and (in the case of movements) “significant identity links.”^25^ In a political economy marked by global concentrations of corporate and oligarchic power, the formation of “strategic partnerships with a wide variety of actors/organizations [with] a vested interest” in health equity is pivotal for building sufficient power to advance lasting change.^26^

The third element of power building indicated in Figure 1 involves strategically negotiating local, state, and national political institutions to ensure that the countervailing power fostered through organizing and magnified through movements and coalitions, isn’t stymied by antimajoritarian rules (e.g. filibuster), partisan discord, or other perennial threats to equity and democracy in the U.S. (e.g., federalism).^27^ For example, the temporary expansion of the Child Tax Credit (CTC) implemented under the American Rescue Plan kept millions of American families out of poverty during the Covid-19 pandemic.^28^ Available evidence suggests that the CTC helped families access basic resources necessary for health and well-being.^29^ Though there were significant nationwide organizing and advocacy efforts devoted to continuing the expanded CTC, it was not made permanent.^30^ This is an indicator of insufficient power among the most affected populations, but it also implicates the constraints of antimajoritarian political institutions like the Senate and destructive political processes like negative partisanship.^31^

Building power requires organizing people and developing coalitional and movement politics in ways that are strategically designed to navigate, negotiate, and ultimately overcome such institutional constraints. For example, in 2017, New York City substantially expanded the civil legal rights of its denizens by passing a law that guaranteed an attorney to every low-income tenant facing eviction.^32^ This policy helped to curb evictions, an important driver of health inequity.^33^ Initially, city officials and other elites did not want to commit the resources to provide legal counsel for NYC tenants facing eviction. However, over a period of four years, a combination of grassroots political organizing, intensive coalition building, and strategic maneuvering of political institutions (e.g., the NYC mayor and city council) led to the passage of an unprecedented “right to counsel” law that sparked similar legislation across the country.^34^ This is one example. Organizing, coalition building, and strategizing to build power can take many forms (too many to enumerate here) and are most clearly conceived in relation specific goals, policies, actors, and outcomes.

## Breaking Power

Breaking power is the second facet of the struggle to achieve health justice. Breaking power involves weakening and destabilizing power relations that undergird health inequity. Such power relations are diffuse, dynamic, entrenched, and often obscured. They implicate a shifting cast of political actors across time, place, and policy arenas. Ideally, efforts to break power should involve those who are most harmed by its deployment. At the same time, when possible, breaking power can occur in coalition with a maximally wide range of actors, including those who may themselves hold kinds of power that need to be broken. Admittedly, the mechanisms for breaking power are difficult to identify and less well investigated by scholars. In this article, I emphasize breaking power through: 1) minimizing profit; 2) administrative regulation and enforcement; and 3) strategic institutional negotiation. These mechanisms are neither exhaustive nor comprehensive. Below, I offer rudimentary thoughts on each, with the caveat that there is an acute need for deeper study of pathways for breaking entrenched power.

Minimizing the profits generated by health inequity is an important mechanism for breaking the power of those with vested interests in maintaining or exacerbating the status quo. So long as health inequity is profitable, elite economic actors to whom such profits accrue will be incentivized to produce and reproduce the systems and practices driving inequity. Admittedly, it is not easy to pinpoint the profits that stem from health injustice. Yet, there is ample evidence that the privatization and commodification of healthcare yields substantial profit to a relatively small cadre of economic elites.^35^ Moreover, because economic and political power are closely tethered in the U.S., the same economic elites that benefit from health inequity exert an outsized influence on politics. This makes curtailing those who benefit from health inequity vital for both health justice and democracy. But doing so is tough. Central institutional features of the U.S. political system empower wealthy Americans and corporate interests.^36^ Moreover, cultural, and ideological attachments to the free market make it difficult to place limits on profiteering in healthcare, housing, and many other policy arenas. This is why regulating the costs of life saving health resources (e.g., prescription medication) or health sustaining material needs (e.g., housing, food) is a heavy political lift. Nonetheless, forging politics and designing policy in ways that erode the revenue garnered from health inequity is one pathway for altering the existing balance of power in the struggle for health justice.

Though breaking power through profit minimization, administrative regulation, and strategic negotiation are neither common nor easy, these mechanisms (at least conceptually) lie at the core of enduring political efforts related to antitrust policies and regulating the cost of healthcare. State and federal efforts to cap the cost of insulin — a lifesaving drug — while not deliberately aimed at breaking power, exemplify the kind of step that can begin to break power if fashioned more intentionally and systematically.

Administrative regulation and enforcement are related and somewhat overlapping mechanisms for curbing the power of actors who gain from health inequity. The administrative state has significant capacity to regulate corporate actors, especially (but not exclusively) in relation to health and healthcare.^37^Agencies like the Federal Drug Administration (FDA), the Department of Agriculture (USDA), and the Environmental Protection Agency (EPA) can influence the costs, quality, and availability of medications, food, and even safe (e.g., non-toxic) living environments. These agencies are often far removed from the reach and oversight of ordinary Americans, but closely connected to corporate or other elite interests.^38^ Breaking the power of such interests requires reorienting regulatory agencies whenever possible, such that they have closer connection to the populations and communities most directly affected by regulatory decisions and are held strictly accountable for their relationships to corporate actors.

Finally, strategic institutional negotiation is as important for breaking power as it is for building it. When the former is the aim, the purpose and nature of strategic maneuvering is different, but still critical. Surfacing economic interests that threaten health equity, tracing their political investments, identifying the institutional processes through which they most frequently operate, and undermining those processes in deliberate ways are all part and parcel of the kind of strategic institutional negotiation that might (over the long term) break power in ways that advance health justice.

Though breaking power through profit minimization, administrative regulation, and strategic negotiation are neither common nor easy, these mechanisms (at least conceptually) lie at the core of enduring political efforts related to antitrust policies and regulating the cost of healthcare. State and federal efforts to cap the cost of insulin — a lifesaving drug — while not deliberately aimed at breaking power, exemplify the kind of step that can begin to break power if fashioned more intentionally and systematically.

## Conclusion

In a widely cited piece focused on health politics, Bambra, Fox, and Scott-Samuel incisively noted that “health is political because power is exercised over it as part of a wider economic, social, and political system. Changing this system requires…political struggle.”^39^ This essay builds on their logic by sketching the contours of two basic elements of such struggle: building power and breaking power. Building power is necessary for those who are disadvantaged by the status quo of health inequity. Breaking power is essential for mitigating the influence of elite interests who reap benefits from health inequity. Both processes represent essential steps on the path to health justice. Though I delineate specific mechanisms for building and breaking power, I necessarily leave many details unaddressed, and many particulars underspecified. I do so because the conceptual, empirical, strategic work necessary for crafting a vision of health justice through the lens of power remains largely before us. This essay is meant to provoke thought and generate ideas towards these ends.
